# Comparison of various surgical incisions in parotidectomy: A systematic review and network meta-analysis

**DOI:** 10.3389/fonc.2022.972498

**Published:** 2022-08-05

**Authors:** Siyue Yin, Yanxun Han, Yuchen Liu, Bangjie Chen, Ziyue Fu, Shuyan Sheng, Jianpeng Wang, Chuanlu Shen, Xinyi Wang, Yiwen Jia

**Affiliations:** ^1^ Department of Oncology, The First Affiliated Hospital of Anhui Medical University, Hefei, China; ^2^ Anhui Medical University, Hefei, China; ^3^ Department of Otolaryngology, Head and Neck Surgery, The First Affiliated Hospital of Anhui Medical University, Hefei, China; ^4^ Department of Gastroenterology, The Third Affiliated Hospital of Anhui Medical University (Hefei first people’s Hospital), Hefei, China

**Keywords:** parotidectomy, surgical incision, Bayesian network meta-analysis, modified Blair incision, modified facelift incision, retroauricular hairline incision, V-shaped incision

## Abstract

**Background:**

This network meta-analysis aimed to comprehensively compare the operative and postoperative outcomes of different parotidectomy incisions.

**Methods:**

Embase, PubMed, Web of Science, and Cochrane Central Register of Controlled Trials were searched up to April 2022. A complete Bayesian network meta-analysis was performed using the Markov Monte Carlo method in OpenBUGS.

**Results:**

Seventeen studies with 1609 patients were included. Thirteen were retrospective cohort studies, three were prospective cohort studies, and one was a randomized controlled study. The quality of evidence was rated as very low in most comparisons. The incision satisfaction score of the modified facelift incision (MFI), retroauricular hairline incision (RAHI), V-shaped incision (VI) were higher than that of the modified Blair incision (MBI) (MBI vs. MFI: mean difference [MD] -1.39; 95% credible interval [CrI] -2.23, -0.57) (MBI vs. RAHI: MD -2.25; 95% CrI -3.40, -1.12) (MBI vs. VI: MD -2.58; 95% CrI -3.71, -1.46); the tumor size treated by VI was smaller than that by MBI (MD 5.15; 95% CrI 0.76, 9.38) and MFI (MD 5.16; 95% CrI 0.34, 9.86); and the risk of transient facial palsy in the MFI was lower than that in the MBI (OR 2.13; 95% CrI 1.28, 3.64). There were no differences in operation time, drainage volume, wound infection, hematoma, salivary complications, Frey syndrome, or permanent facial palsy between incision types.

**Conclusion:**

The traditional MBI is frequently used for large tumor volumes, but the incision satisfaction score is low and postoperative complication control is poor. However, emerging incisions performed well in terms of incision satisfaction scores and control of complications. More randomized controlled trials are needed to compare the different parotidectomy incisions. Patients should be fully informed about the characteristics of each incision to make the most informed decision, along with the physician’s advice.

**Systematic Review Registration:**

PROSPERO, identifier CRD42022331756

## 1 Introduction

The parotid glands, being the largest pair of salivary glands in the human body, are the location of approximately 80% of salivary gland cancer (1). According to the International Agency for Research on Cancer, there were 53,583 new cases of salivary gland cancer globally in 2020, accounting for 0.3% of all cancers (2). Most parotid tumors are benign, and parotidectomy is the preferred treatment option because of recurrence and potential malignant transformation (3, 4). Since the classic cervicomastoidfacial incision was proposed by Blair in 1912, the operative approach for parotid gland resection has undergone continuous improvement and innovation (5). Endoscopy- and robot-assisted parotidectomy techniques have also progressed in recent years, but their safety and ease of use need to be further proven in practice.

Currently, four incision types are commonly used for parotidectomy. The modified Blair incision (MBI) is the most widely used surgical incision in the clinic, while the modified facelift incision (MFI), retroauricular hairline incision (RAHI), and V-shaped incision (VI) are becoming increasingly prevalent. A large-scale surgical incision allows for full exposure of the parotid gland tissue to minimize facial nerve injury, but the ensuing huge facial scar inevitably inflicts a psychological load on the patient (6, 7). In contrast, smaller incisions with better cosmetic results require persuasive data representation to control complications.

There has been ongoing discussion regarding the different incision types for parotidectomy. Unfortunately, the number of relevant meta-analyses is limited (8). This study compared four incision options for parotidectomy based on a Bayesian network meta-analysis with the aim of providing evidence for surgical and patient incision selection.

## 2 Methods

### 2.1 Search strategy

This study was registered *a priori* with PROSPERO (CRD42022331756). We conducted a systematic literature search of Embase, PubMed, Web of Science, and Cochrane Central Register of Controlled Trials in April 2022 and were not restricted with regard to publication language and date. The complete search strategy is presented in **Supplementary Material**. We also reviewed the references of the included articles to identify additional potential studies. Because all analyses were based on previously published studies, ethical approval and patient consent were not required.

### 2.2 Study selection

**Figure 1:**
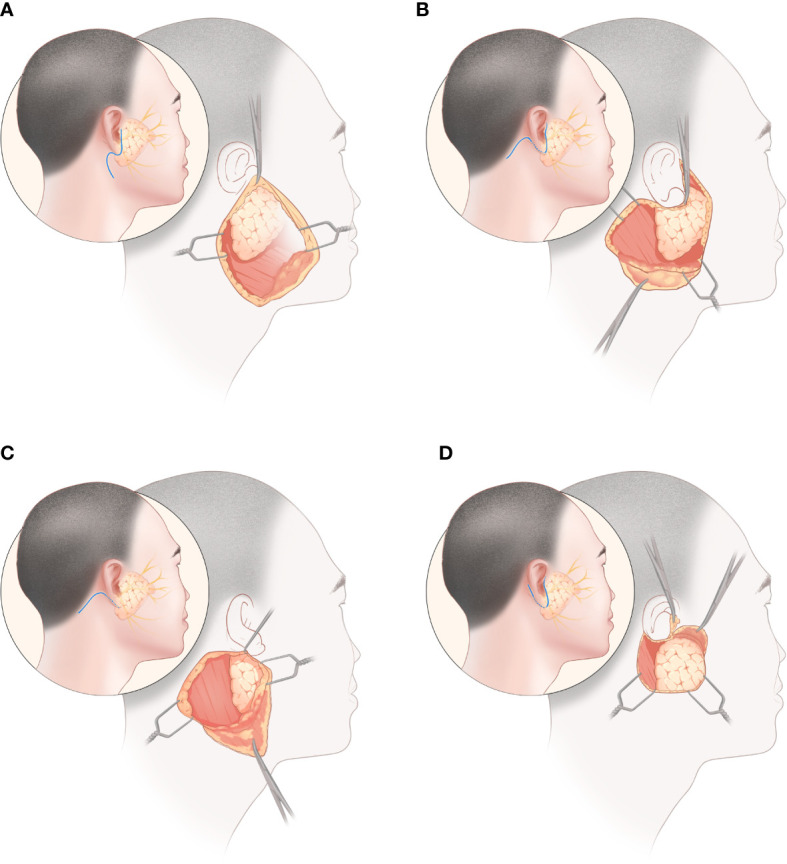
Parotidectomy *via* four incisions: **(A)** modified Blair incision, **(B)** modified facelift incision, **(C)** retroauricular hairline incision, **(D)** V-shaped incision.

Following the Preferred Reporting Items for Systematic Reviews and Meta-Analysis guidelines, studies were included based on the population, intervention, comparison, outcome, and study design (PICOS). The PICOS components of this study were as follows: P (patients who underwent parotidectomy with speculated benign parotid tumors on preoperative examination), I (use of MBI **[** **Figure 1A** **]**, MFI **[** **Figure 1B** **]**, RAHI **[** **Figure 1C** **]**, or VI **[** **Figure 1D** **]** in parotidectomy), C (pairwise comparisons between the four incisions), O (intraoperative and postoperative parameters, including operation time, incision satisfaction score, drainage volume, permanent facial palsy, bleeding volume, transient facial palsy, Frey syndrome, salivary complications, wound infection, and hematoma), and S (randomized clinical trials [RCTs] or original research articles with prospective or retrospective designs).

The inclusion criteria were as follows: 1) RCTs or original research articles with prospective or retrospective designs, 2) articles that included patients who underwent parotidectomy and who had speculated benign parotid tumors by preoperative examination, and 3) studies that reported the outcome of parotidectomy and included at least one required outcome measure. The exclusion criteria were as follows: 1) studies using endoscopes or robots to assist with surgery, 2) studies with no control group, 3) studies related to flap or fascia reconstruction, 4) articles not published in English, and 5) review articles, short reports, and letters to the editor.

### 2.3 Data extraction and quality assessment

Data were independently extracted by two investigators. All divergences that arose throughout the procedure were reviewed by a third investigator, and a decision was made. The extracted data included the name of the first author, year of publication, country, study type, age, sex, duration of follow-up, surgical approach, tumor size, operation time, and postoperative outcomes. The primary outcomes were incision satisfaction score, operation time, drainage volume, and permanent facial palsy. Secondary outcomes were tumor size, transient facial palsy, Frey syndrome, salivary complications, wound infection, and hematoma. If relevant data were missing, an approximate formula was used for the calculation. The quality of the included RCTs was evaluated using the Cochrane Collaboration Tool, while the Newcastle-Ottawa scale (NOS) and the risk of bias in non-randomized studies of interventions (ROBINS-I) were used to assess the quality of the cohort studies. The evaluation criteria for the RCT tool included the randomization procedure, allocation concealment, baseline comparability of the research groups, blinding, and completeness of follow-up (9). NOS evaluates and scores study bias out of 9 points, including 4 for patient selection, 2 for research group comparability, and 3 for outcome evaluation. The ROBINS-I assesses bias due to confusion, subject selection, intervention classification, deviations from expected interventions, missing data, outcome measures, and reported outcome selection.

### 2.4 Statistical analysis

This network meta-analysis was conducted according to the Preferred Reporting Items for Systematic Reviews and Meta-Analysis guidelines (10). For continuous variables, the mean difference (MD) was calculated. As the variables in the categorical data were all adverse event outcomes and the positive rate was low, the odds ratio (OR) was used to calculate the effect size. For zero positive event outcomes, 0 was replaced by 0.5 to prevent a large confidence interval (11). A pairwise comparison meta-analysis was performed to obtain direct comparison results. To visualize all head-to-head comparisons for each outcome, we created network plots. Our study was based entirely on the random effects of the Bayesian approach and was analyzed using the Monte Carlo Markov chain (MCMC) method in OpenBUGS Version 3.2.3. The auxiliary statistical analysis and mapping were performed using software R 4.1.3 (main packages including gemtc and rjags) and Stata V.14. The fit of the model was verified using totresdev, and convergence was ensured using trace plots, Autocorr, Brooks-Gelman-Rubin diagnostic diagrams, and Potential Scale Reduction Factor (PSRF). The deviance information criterion (DIC) of the consistent and inconsistent models was compared to select a better model (12). If the inconsistent model had a better fit (low DIC value), the results were interpreted with caution (13). League tables were used to show the pooled comparisons for each outcome. We tested the overall heterogeneity of the outcomes and compared local inconsistencies using the node-splitting method. The evaluation criteria for statistical heterogeneity were as follows: I^2^ index values below 25% were considered as low heterogeneity, 50% as moderate heterogeneity, and 75% as high heterogeneity. Statistical significant was set at P<0.05. The surface under the cumulative ranking (SUCRA) was used to rank the inspected interventions (14). Furthermore, we evaluated publication bias using a funnel plot for outcomes in more than 10 studies. Finally, we used the network meta-analysis recommendations for grading and developed GRADE to assess the certainty of evidence (15).

## 3 Results

### 3.1 Search results and methodological quality

**Figure 2:**
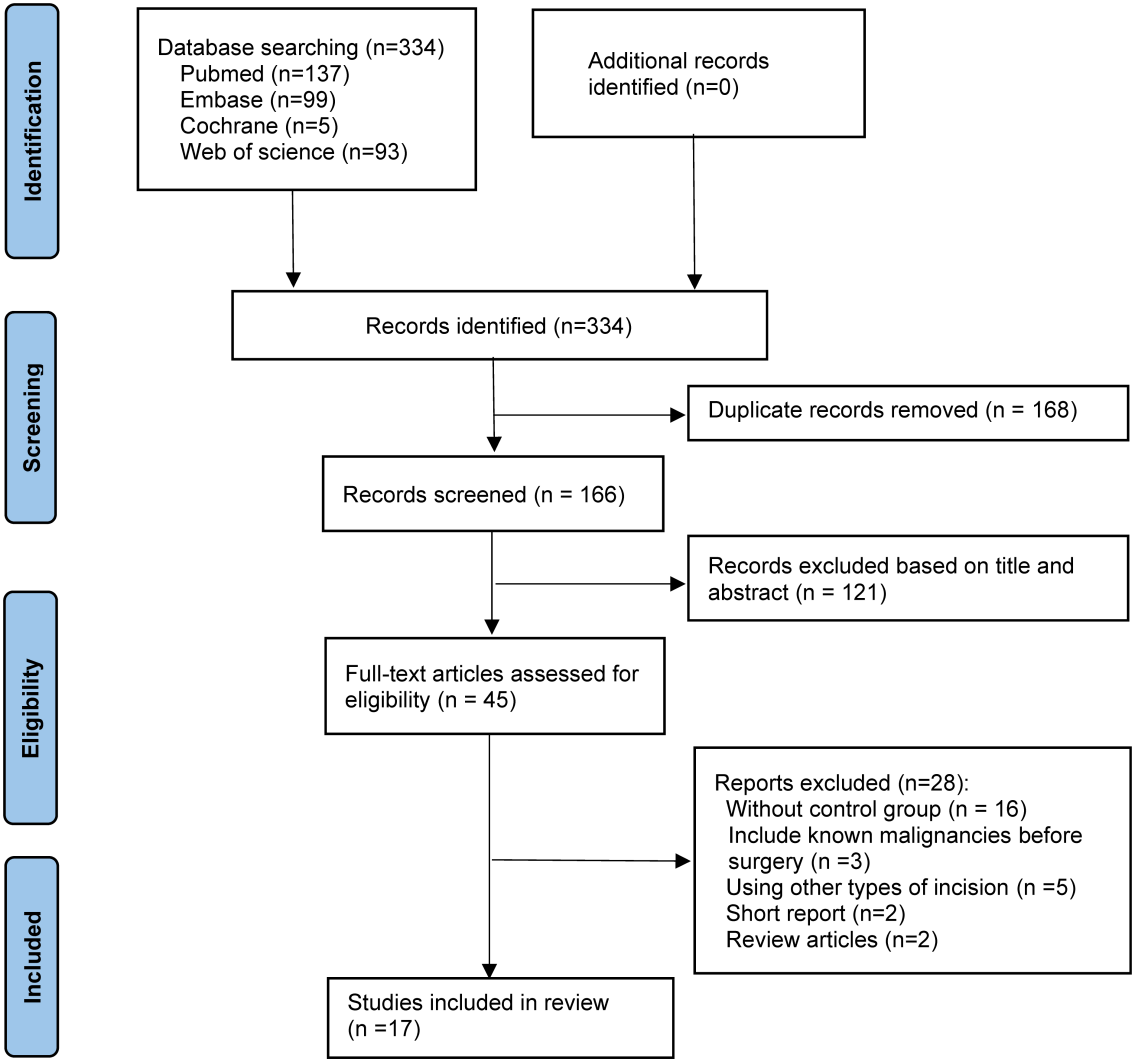
The Preferred Reporting Items for Systematic Reviews and network meta-analyses checklist (PRISMA-NMA) diagram.

The selected databases were searched for 334 potentially related articles. Following the removal of duplicate studies, the titles and abstracts of 166 selected studies were examined and 121 unqualified papers were eliminated. After reading the full text, 1609 patients were included across 17 qualified articles, including one RCT, 13 retrospective cohort studies, and three prospective cohort studies. **Figure 2** shows literature selection procedure in this study.

The baseline characteristics of the 17 types of research included in the network meta-analysis are presented in **Table 1** (16–32). In our analysis, 14 of the studies were two-arm trials and three were three-arm trials involving four different surgical procedures. Nine cohort studies were considered to be of high quality, with NOS scores of 7 or greater. Specific scores are presented in **Table 1**. **Table 2** shows the findings of the bias risk assessment for cohort studies using the ROBINS-I, with eight studies having a low overall bias. The results of RCT evaluated by the Cochrane Collaboration Tool are shown in **Table 3**.

**Table 1:** Characteristics and NOS quality assessment of the included studies.

Author, year, country	Study design	Surgical procedure	No. of patient	Age (years)	Gender (M/F)	Follow-up (months)	Tumor size (mm)	Newcastle-Ottawa Scale			
								Selection	Comparability	Outcome	Total (9☆)
Terris (16), USA	RCS	MBI	15	40.3 ± 24.6	5/10	7.7 ± nr	nr	☆☆☆	☆	☆☆	6
		MFI	17	40.3 ± 12.3	1/16	8.1 ± nr					
Roh (17) Korea	RCS	MBI	49	50.5 ± 15.7	23/26	48 ± 23	29 ± 19	☆☆☆☆	☆☆	☆☆☆	9
		MFI	52	48.4 ± 14.6	24/28	47 ± 22	27 ± 18				
Wasson (18), UK	RCS	MBI	59	51 ± nr	29/30	≥6	nr	☆☆☆☆	☆	☆	6
		MFI	20	44 ± nr	11/9						
Bianchi (19), Italy	RCS	MBI	35	nr	nr	≥18	nr	☆☆☆	☆	☆☆☆	7
		MFI	48								
Lee (20), Korea	RCS	MBI	162	45.82 ± 18.44	90/72	8.98 ± nr	26.49 ± 11.94	☆☆☆☆	☆	☆☆	7
		MFI	182	44.12 ± 16.76	51/131		23.76 ± 9.98				
Zhi (21), China	RCS	MBI	20	49 ± nr	nr	36 ± 0	nr	☆☆☆	☆	☆☆☆	7
		MFI	18	45 ± nr							
Graciano (22) Brazil	RCS	MBI	30	47.3 ± nr	21/9	nr	48.12 ± nr	☆☆☆	☆	☆☆	6
		MFI	30	34.93 ± nr	11/19		34.29 ± nr				
Kim (23), Korea	RCS	MBI	16	45 ± nr	6/10	29 ± NA	27.1 ± nr	☆☆☆	☆☆	☆☆☆	8
		MFI	24	51 ± nr	9/15		27.4 ± nr				
		RAHI	33	46 ± nr	14/19		27.8 ± nr				
Bulut (24) Germany	RCS	MBI	24	43 ± nr	5/19	97 ± NA	31 ± nr	☆☆☆☆	☆	☆☆☆	8
		MFI	24	43 ± nr	5/19		29 ± nr				
Wu (25), China	RCS	MBI	28	47.2 ± 14.1	14/14	25 ± 0	22 ± 9	☆☆☆	☆☆	☆☆	7
		RAHI	36	48.1 ± 18.0	22/14		24 ± 9				
Xu (26), China	PCS	MBI	35	41.66 ± 13.18	17/18	48 ± nr	37.2 ± 6.9	☆☆☆	☆	☆☆☆	7
		MFI	36	39.46 ± 11.18	14/22		35.7 ± 6.5				
Zheng (27), China	PCS	MBI	23	37.5 ± 8.9	11/12	19.2 ± 2.8	25.1 ± 5	☆☆☆	☆	☆☆	6
		VI	23	36.2 ± 8.7	10/13	18.7 ± 2.6	23 ± 6				
Jo, Korea	PCS	MBI	40	51.1 ± 17	19/21	nr	24.7 ± 7.9	☆☆☆☆	☆	☆	6
		VI	34	46.3 ± 13.4	13/21		21.4 ± 5.8				
Ahn (28), Korea	RCS	MFI	122	53.5 ± 14.8	71/51	nr	28 ± 11	☆☆☆☆	☆	☆☆	7
		RAHI	50	51.8 ± 17.7	24/26		27 ± 10				
		VI	41	42.1 ± 14.5	12/29		19 ± 5				
Li (29), China	RCT	MBI	20	43.35 ± 8.83	15/5	nr	22.5 ± nr	–	–	–	–
		MFI	20	45.95 ± 8.16	16/4		17 ± nr				
		VI	20	43.40 ± 9.89	16/4		18 ± nr				
Zhang, China	RCS	MBI	36	nr	23/13	6	nr	☆☆☆	☆☆	☆	6
		MFI	32		nr						
Chen (30), China	RCS	MFI	29	56 ± 11.86	16/13	nr	27.7 ± 9.9	☆☆☆	☆	☆☆	6
		RAHI	19	39 ± 14.49	6/13		24.3 ± 9				
Matsumoto (21), Japan	RCS	MBI	97	50.71 ± 15.08	35/62	nr	26.36 ± 10.77	☆☆☆	☆☆	☆☆	7
		MFI	78	51.99 ± 13.53	29/49	nr	25.78 ± 11.85				

RCS, retrospective cohort study; PCS, prospective cohort study; RCT, randomized controlled trial; MBI, modified Blair incision; MFI, modified facelift incision; RAHI, retroauricular hairline incision; VI, V-shaped incision; nr, not reported. The number of * corresponds to the score.

**Table 2:** Risk of bias assessment in cohort studies by ROBINS-I.

Study	Year	Bias due to confounding	Bias in selection of participants into the study	Bias in classification of interventions	Bias due to deviations from intendedinterventions	Bias due to missing data	Bias in measurement of outcomes	Bias in selection of the reported result	Overall
Terris	16	Moderate	Moderate	Low	Low	Low	Moderate	Low	Moderate
Roh	17	Low	Low	Low	Low	Low	Low	Low	Low
Wasson	18	Critical	Low	Low	Low	Low	Low	Low	Critical
Bianchi	19	Low	Moderate	Low	Low	Moderate	Low	Low	Moderate
Lee	20	Low	Low	Low	Low	Low	Low	Low	Low
Zhi	21	Low	Low	Low	Low	Low	Low	Low	Low
Graciano	22	Moderate	Moderate	Low	Low	Low	Low	Low	Moderate
Kim	23	Low	Low	Low	Low	Low	Low	Low	Low
Bulut	24	Low	Low	Low	Low	Moderate	Low	Low	Moderate
Wu	25	Low	Low	Low	Low	Low	Low	Low	Low
Xu	26	Low	Low	Low	Low	Low	Low	Low	Low
Zheng	27	Moderate	Moderate	Low	Low	Low	Low	Low	Moderate
Jo		Low	Low	Low	Low	Low	Low	Low	Low
Zhang		Critical	Moderate	Low	Low	Low	Low	Low	Critical
Ahn	28	Moderate	Low	Low	Low	Low	Low	Low	Moderate
Chen	30	Low	Moderate	Low	Low	Low	Low	Low	Moderate
Matsumoto	21	Low	Low	Low	Low	Low	Low	Low	Low

**Table 3:** Risk of bias of one RCT with Cochrane Collaboration tools.

Risk of bias	Li et al., 2020, China
Random sequence generation (selection bias)	Quote: “Patients meeting the inclusion criteria were randomly divided into three incision groups by lottery” Method of random sequence generation can produce comparison groups.
Allocation concealment (selection bias)	Impossible due to nature of surgery and peroperative consent.
Blinding of participants and personnel (performance bias)	Impossible due to nature of surgery and peroperative consent.
Blinding of outcome assessme (detection bias)	Impossible due to nature of surgery and peroperative consent.
Incomplete outcome data (attrition bias)	There was no incomplete or missing data.
Selective reporting (reporting bias)	Consistency between outcome measure in methods and results.
Other bias	There were no other sources of bias

Red, yellow and green correspond to a high risk, unknown risk and low risk of bias, respectively.

### 3.2 Traditional meta-analyses

**Figure 3:**
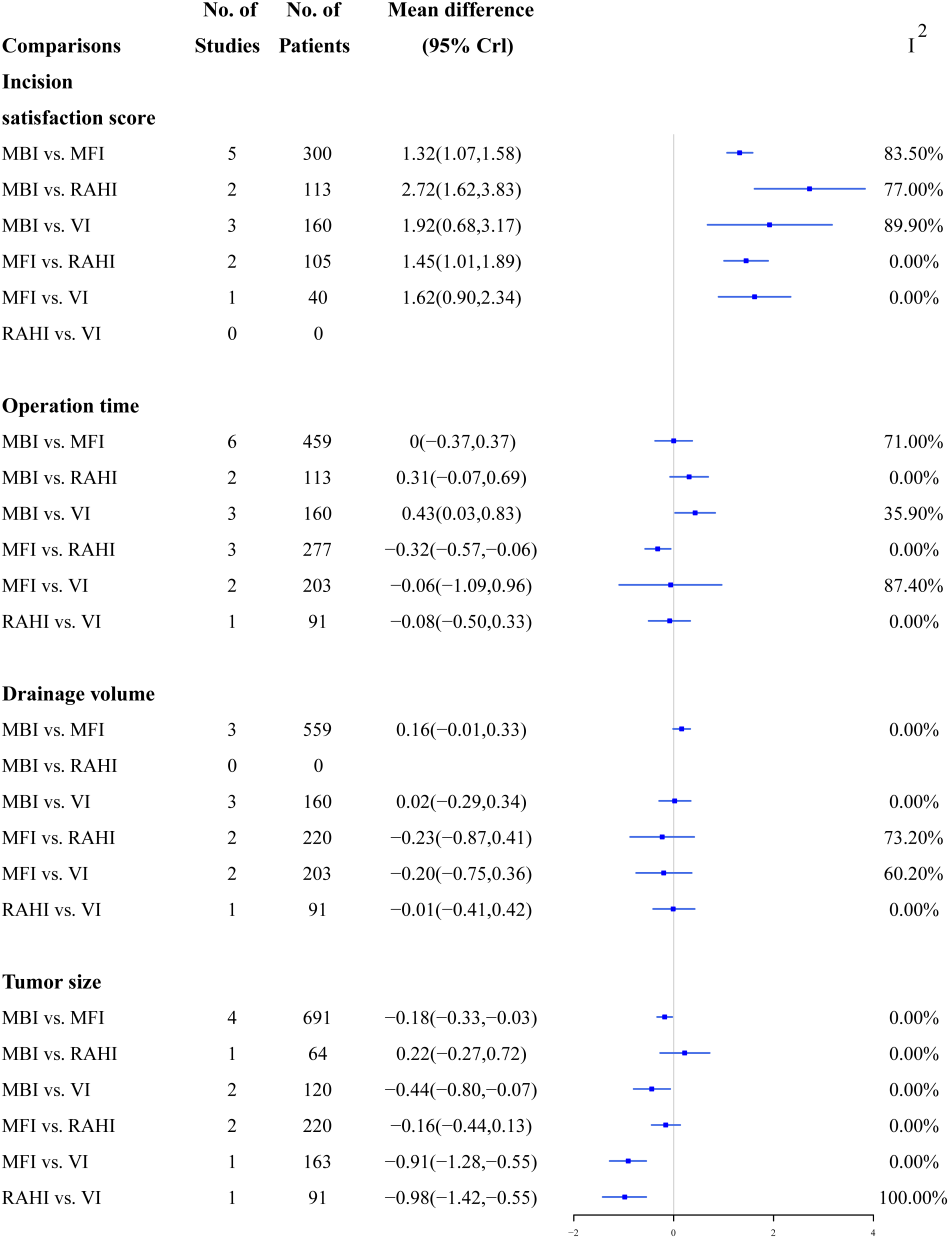
A direct comparison forest map of continuous outcomes.

**Figure 4:**
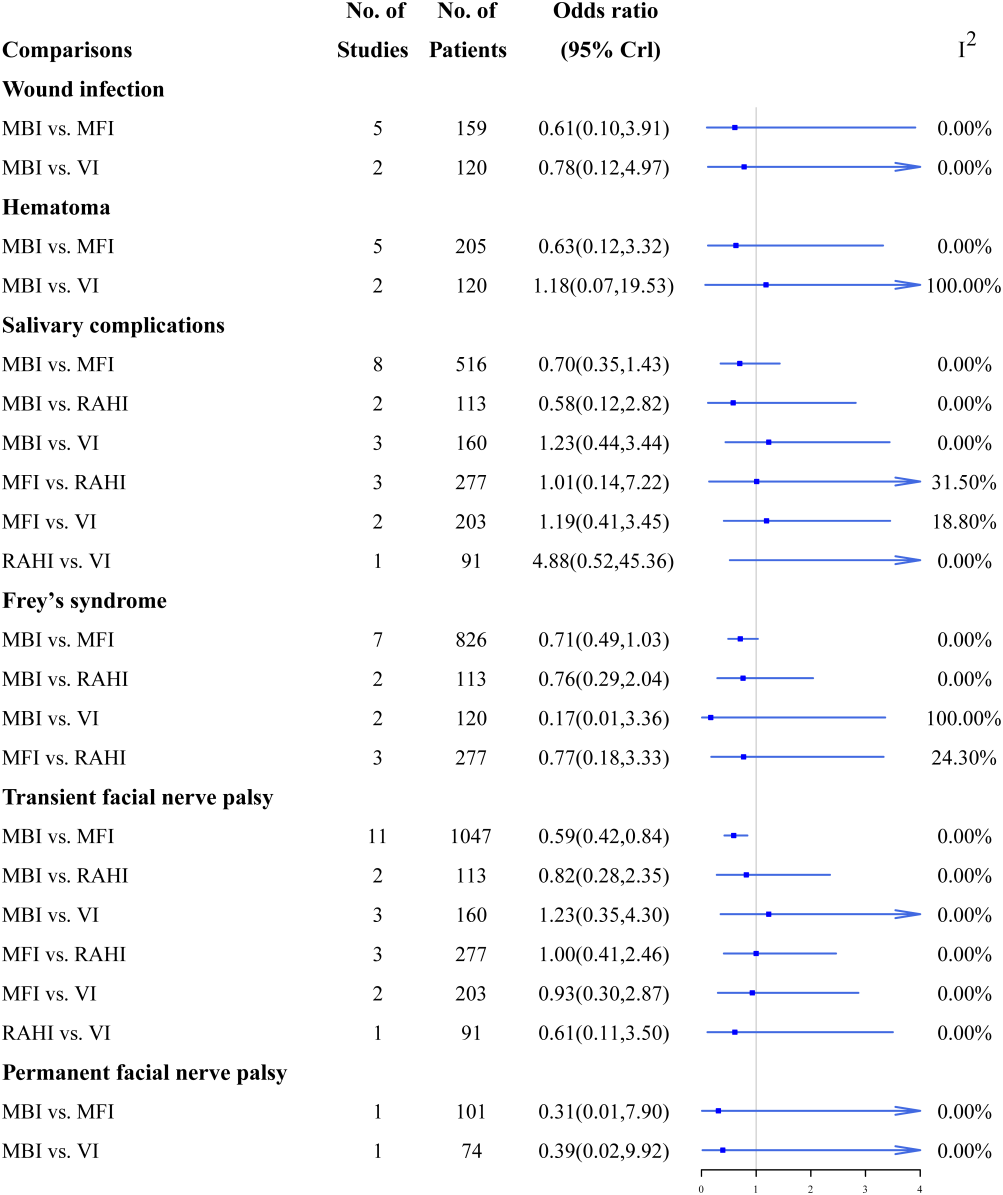
A direct comparison forest map of dichotomous outcomes.

**Figures 3**, **4** summarize the direct comparison results of the pairwise meta-analyses of continuous and dichotomous outcomes from the 17 studies, respectively. The MFI, RAHI and VI had significantly higher incision satisfaction scores than the MBI; the RAHI and VI had significantly higher incision satisfaction scores than the MFI; however, no study has directly compared the RAHI and VI. VI required substantially longer time to operate than MBI, whereas MFI lasted significantly longer than RAHI. MBI had a significantly larger tumor size than MFI, whereas VI was significantly smaller than the other three incisions. The incidence of transient facial palsy was significantly higher only in the MBI group when compared with the MFI group, and there was no statistical significance in a pairwise direct comparison of other complications. Overall, the heterogeneity was low, although several groups had high values, reflecting differences in surgical skills among physicians or the small number of studies included in these pairwise comparisons.

### 3.3 Bayesian network meta-analyses

**Figure 5:**
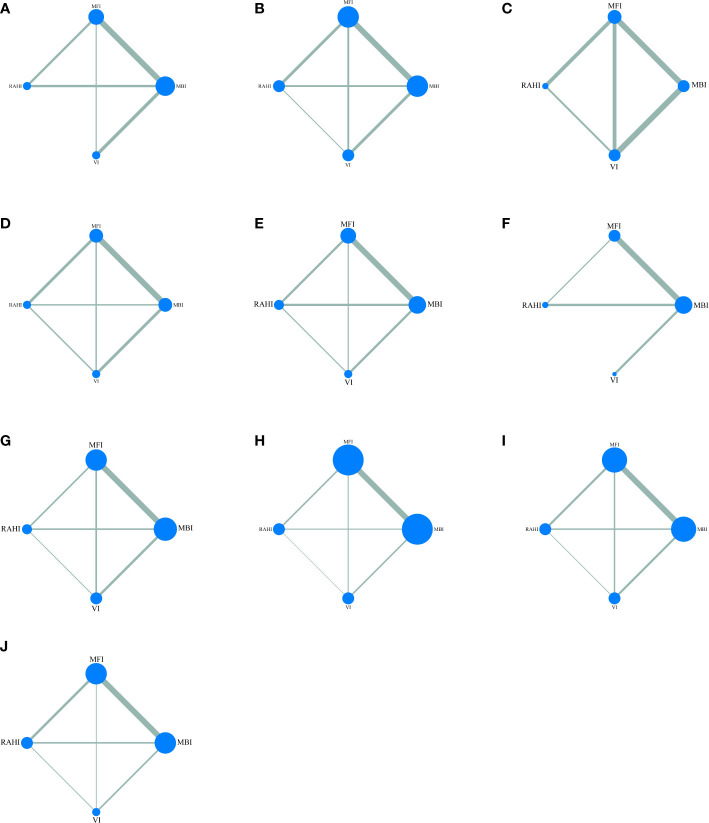
Network maps of all outcomes: **(A)** incision satisfaction score, **(B)** operation time, **(C)** drainage volume, **(D)** tumor size, **(E)** hematoma, **(F)** wound infection, **(G)** permanent facial palsy, **(H)** transient facial palsy, **(I)** salivary complications, **(J)** Frey syndrome.

**Figure 5** shows the network relationships between the different incisions. The area of each circle denotes the number of patients included, and the thickness of the lines linking the two surgical incisions represents the number of articles. **Table 4** displays the pooled comparison findings, with the statistically significant values highlighted in bold.

**Table 4:** League table of primary outcomes.

MBI				A
-0.76 (-2.19,0.66)	**MFI**			
-1.95 (-4.08,0.17)	-1.18 (-3.30,0.93)	**RAHI**		
-2.47 (-4.51,-0.43)	-1.70 (-4.04,0.62)	-0.52 (-3.39,2.35)	**VI**	
**MBI**				B
-1.67 (-11.49,10.39)	**MFI**			
-0.40 (-13.52,14.08)	1.30 (-12.60,14.16)	**RAHI**		
-3.53 (-17.91,9.81)	-1.86 (-18.42,11.48)	-3.12 (-21.20,12.57)	**VI**	
**MBI**				C
-3.22 (-15.16,5.55)	**MFI**			
7.54 (-13.56,22.30)	10.99 (-6.35,24.47)	**RAHI**		
0.36 (-10.68,11.63)	3.67 (-7.12,17.53)	-7.15 (-22.19,14.14)	**VI**	
**MBI**				D
1.04 (0.30,7.49)	**MFI**			
0.56 (0.11,25.13)	0.37 (0.07,18.85)	**RAHI**		
0.60 (0.13,11.98)	0.36 (0.07.10.17)	0.15 (0.03,20.62)	**VI**	

Values of A, B, and C are expressed as mean difference (MD) and 95% credible intervals (95% CrI).

Values of D is expressed as odds ratio (OR) and 95% credible intervals (95% CrI).

A incision satisfaction score, B operation time, C drainage volume, D permanent facial palsy.

The bold values indicate that the comparison between the two is statistically significant.

#### 3.3.1 Incision satisfaction score

Nine studies including 585 patients provided data on incision satisfaction scores. Meta-analysis of pooled network showed similar MDs when comparing MFI vs. RAHI (MD -0.85; 95% credible interval [CrI] -2.00, 0.28), MFI vs. VI (MD -1.18; 95% CrI -2.49, 0.11), RAHI vs. VI (MD -0.33; 95% CrI -1.88, 1.23), while MBI vs. MFI (MD -1.39; 95% CrI -2.23, -0.57), MBI vs. RAHI (MD -2.25; 95% CrI -3.40, -1.12), and MBI vs. VI (MD -2.58; 95% CrI -3.71, -1.46) were signifificant (**Table 4A**). No statistical difference was observed between direct and indirect comparisons (MFI vs. MBI, p=0.08; RAHI vs. MBI, p=0.36; RAHI vs. MFI, p=0.21; VI vs. MFI, p=0.14). The overall heterogeneity was low (I^2 =^ 6%). The SUCRA rankings were 0.1% for MBI, 36% for MFI, 75% for RAHI, and 88% for VI.

#### 3.3.2 Operation time

Eleven studies involving 957 patients reported the operation time. Meta-analysis of the pooled network showed similar MDs when comparing MBI vs. MFI (MD -1.67; 95% CrI -11.49, 10.39), MBI vs. RAHI (MD -0.40; 95% CrI -13.52, 14.08), MBI vs. VI (MD -3.53; 95% CrI -17.91, 9.81), MFI vs. RAHI (MD 1.30; 95% CrI -12.60, 14.16), MFI vs. VI (MD -1.86; 95% CrI -18.42, 11.48), RAHI vs. VI (MD -3.12; 95% CrI -21.20, 12.57) (**Table 4B**). No statistical difference was observed between the direct and indirect comparisons (MFI vs. MBI, p=0.09; RAHI vs. MBI, p=0.52; VI vs. MBI, p=0.09; RAHI vs. MFI, p=0.29; VI vs. MFI, p=0.35; VI vs. RAHI, p=0.40). The overall heterogeneity was low (I^2 =^ 13%). The SUCRA rankings were 63% for MBI, 46% for MFI, 58% for RAHI, and 33% for VI.

#### 3.3.3 Drainage volume

Seven studies with a total of 960 patients reported the drainage volume. Meta-analysis of the pooled network showed similar MDs when comparing MBI vs. MFI (MD -3.22; 95% CrI -15.16, 5.55), MBI vs. RAHI (MD 7.54; 95% CrI -13.56, 22.30), MBI vs. VI (MD 0.36; 95% CrI -10.68, 11.63), MFI vs. RAHI (MD 10.99; 95% CrI -6.35, 24.47), MFI vs. VI (MD 3.67; 95% CrI -7.12, 17.53), RAHI vs. VI (MD -7.15; 95% CrI -22.19, 14.14) (**Table 4C**). No statistical difference was observed between the direct and indirect comparisons (MFI vs. MBI, p=0.41; V vs. MBI, p=0.35; VI vs. MFI, p=0.92; VI vs. RAHI, p=0.70). The overall heterogeneity was low (I^2 =^ 14%). The SUCRA rankings were 50% for MBI, 15% for MFI, 84% for RAHI, and 51% for VI.

#### 3.3.4 Permanent facial palsy

Eleven studies of 969 patients reported permanent facial palsy. Meta-analysis of the pooled network showed similar ORs when comparing MBI vs. MFI (OR 1.04; 95% CrI 0.30, 7.49), MBI vs. RAHI (OR 0.56; 95% CrI 0.11, 25.13), MBI vs. VI (OR 0.60; 95% CrI 0.13, 11.98), MFI vs. RAHI (OR 0.37; 95% CrI 0.07, 18.85), MFI vs. VI (OR 0.36; 95% CrI 0.07, 10.17), RAHI vs. VI (OR 0.15; 95% CrI 0.03, 20.62) (**Table 4D**). No statistical difference was observed between the direct and indirect comparisons (MFI vs. MBI, p=0.71; RAHI vs. MBI, p=0.90; V vs. MBI, p=0.61; RAHI vs. MFI, p=0.40; VI vs. MFI, p=0.60; VI vs. RAHI, p=0.65). The overall heterogeneity was low (I^2 =^ 0%). The SUCRA rankings were 7% for MBI, 57% for MFI, 61% for RAHI, and 76% for VI.

#### 3.3.5 Secondary outcomes

**Tables 5** and **6** provide a mixed comparison and SUCRA rankings of the secondary outcomes, respectively. Meta-analysis of the pooled network did not show statistically significant OR comparing MBI vs. MFI, MBI vs. RAHI, MBI vs. VI, MFI vs. RAHI, MFI vs. VI, and RAHI vs. VI in terms of hematoma (OR 1.22, 95% CrI 0.35, 9.09; OR 0.52, 95% CrI 0.10, 33.33; OR 0.33, 95% CrI 0.07, 12.50; OR 0.28, 95% CrI 0.05, 16.67; OR 0.15, 95% CrI 0.03, 9.09; OR 0.08, 95% CrI 0.02, 16.67, respectively). The SUCRA rankings were 40% for MBI, 67% for MFI, 56% for RAHI, and 37% for VI. Comparisons of the OR between MBI vs. MFI, MBI vs. RAHI, MBI vs. VI, MFI vs. RAHI, MFI vs. VI, and RAHI vs. VI were also not statistically significant for wound infection (OR 0.84, 95% CrI 0.21, 10.13; OR 0.26, 95% CrI 0.07, 98.14; OR 0.12, 95% CrI 0.09, 17.69; OR 0.10, 95% CrI 0.04, 80.26; OR 0.03, 95% CrI 0.04, 22.54; OR 0.001, 95% CrI 0.01, 49.00, respectively). The SUCRA rankings were 37% for MBI, 54% for MFI, 60% for RAHI, and 49% for VI. In addition, the pooled network meta-analysis did not find statistically significant ORs comparing MBI vs. MFI, MBI vs. RAHI, MBI vs. VI, MFI vs. RAHI, MFI vs. VI, and RAHI vs. VI in terms of salivary complications and Frey syndrome (salivary complications: OR 1.36, 95% CrI 0.73, 2.83; OR 1.60, 95% CrI 0.59, 6.56; OR 0.86, 95% CrI 0.37, 2.57; OR 1.11, 95% CrI 0.42, 4.62; OR 0.59, 95% CrI 0.25, 1.85; OR 0.39, 95% CrI 0.12, 1.99, respectively) (Frey syndrome: OR 1.41, 95% CrI 0.77, 2.63; OR 1.57, 95% CrI 0.66, 5.17; OR 3.11, 95% CrI 0.80, 62.03; OR 1.06, 95% CrI 0.45, 3.55; OR 2.08, 95% CrI 0.53, 42.44; OR 1.57, 95% CrI 0.37, 38.00, respectively). The SUCRA rankings of the former were 26% MBI, 66% MFI, 79% RAHI, and 28% VI, while that of the latter were 9%, 46%, 57%, and 89%, respectively. The overall heterogeneity of the four complications was zero (I^2 =^ 0%). Similarly, pooled network meta-analysis did not demonstrate statistically significant ORs comparing MBI vs. RAHI, MBI vs. VI, MFI vs. RAHI, MFI vs. VI, and RAHI vs. VI in terms of transient facial palsy (OR 1.37, 95% CrI 0.60, 4.00; OR 1.23, 95% CrI 0.50, 4.17; OR 0.69, 95% CrI 0.30, 1.96; OR 0.62, 95% CrI 0.25, 2.13; OR 0.75, 95% CrI 0.26, 3.45, respectively). In contrast, MFI was associated with a statistically significant lower facial palsy compared to MBI (OR, 1.92; 95% CrI, 1.22, 2.94). The SUCRA rankings were 15% for MBI, 82% for MFI, 55% for RAHI, and 49% for VI. Overall heterogeneity was low (I^2 =^ 0). Finally, meta-analysis of pooled networks showed similar MDs in terms of tumor size when comparing MBI vs. MFI (MD -0.01; 95% CrI -3.44, 3.39), MBI vs. RAHI (MD -0.26; 95% CrI -5.04, 4.71), MFI vs. RAHI (MD -0.24; 95% CrI -4.90, 4.61) and RAHI vs. VI (MD 5.41; 95% CrI -0.28, 10.73). However, MBI vs. VI (MD 5.15; 95% CrI 0.76, 9.38), and MFI vs. VI (MD 5.16; 95% CrI 0.34, 9.86) were significant. The SUCRA rankings were 36% for MBI, 35% for MFI, 31% for RAHI, and 98% for VI. Overall heterogeneity was low (I^2 =^ 4).

**Table 5:** League table of secondary outcomes.

MBI				Tumor size
-0.01(-3.44,3.39)	**MFI**			
-0.26 (-5.04,4.71)	-0.24(-4.90,4.61)	**RAHI**		
**5.15(0.76,9.38)**	**5.16(0.34,9.86)**	5.41(-0.28,10.73)	**VI**	
**MBI**				**Hematoma**
1.22(0.35,9.09)	**MFI**			
0.52(0.10,33.33)	0.28(0.05,16.67)	**RAHI**		
0.33(0.07,12.50)	0.15(0.03,9.09)	0.08(0.02,16.67)	**VI**	
**MBI**				**Wound infection**
0.84(0.21,10.13)	**MFI**			
0.26(0.07,98.14)	0.10(0.04,80.26)	**RAHI**		
0.12(0.09,17,69)	0.03(0.04,22.54)	0.001(0.01,49.00)	**VI**	
**MBI**				**Transient facial palsy**
**1.92(1.22,2.94)**	**MFI**			
1.37(0.60,4.00)	0.69(0.30,1.96)	**RAHI**		
1.23(0.50,4.17)	0.62(0.25,2.13)	0.75(0.26,3.45)	**VI**	
**MBI**				**Salivary complications**
1.36(0.73,2.83)	**MFI**			
1.60(0.59,6.56)	1.11(0.42,4.62)	**RAHI**		
0.86(0.37,2.57)	0.59(0.25,1.85)	0.39(0.12,1.99)	**VI**	
**MBI**				**Frey syndrome**
1.41(0.77,2.63)	**MFI**			
1.57(0.66,5.17)	1.06(0.45,3.55)	**RAHI**		
3.11(0.80,62.03)	2.08(0.53,42.44)	1.57(0.37,38.00)	**VI**	

The bold values indicate that the comparison between the two is statistically significant.

**Table 6:** SUCRA of secondary outcomes.

Outcomes (%)Incisions	MBI	MFI	RAHI	VI
tumor size	36	35	31	98
hematoma	40	67	56	37
wound infection	37	54	60	49
transient facial palsy	15	82	55	49
salivary complications	26	66	79	28
Frey syndrome	9	46	57	89

The smaller the tumor size, the greater the SUCRA value.

The lower the incidence of adverse outcomes, the higher the SUCRA value.

### 3.4 Validation and evaluation of models and results

The model fit using the diagnostic approach described in the methodology was good, and there was no evidence that any results were non-MCMC convergent. Furthermore, the node-splitting method did not exhibit any local inconsistencies. By comparing the adjusted funnel plots, no publication bias was observed in operation time, transient facial palsy, permanent facial palsy, or salivary gland complications; however, publication bias was found regarding the incision satisfaction score and Frey syndrome. Funnel plots were not drawn for the other outcomes because fewer than 10 studies were included. The GRADE rating results are listed in **Table 7**. The quality of evidence was rated as very low in most comparisons.

**Table 7:** Quality of evidence for outcomes based on the GRADE method.

Outcomes	Comparison	Study limitations	Imprecision	Inconsistency	Indirectness	Quality of evidence
incision satisfaction score	MBI vs. MFI	Downgraded	Downgraded	No downgraded	No downgraded	Very low
	MBI vs. RAHI	Downgraded	Downgraded	No downgraded	No downgraded	Very low
	MBI vs. VI	Downgraded	No downgraded	No downgraded	No downgraded	Low
	MFI vs. RAHI	Downgraded	Downgraded	No downgraded	No downgraded	Very low
	MFI vs. VI	Downgraded	Downgraded	No downgraded	No downgraded	Very low
	RAHI vs. VI	Downgraded	Downgraded	No downgraded	Downgraded	Very low
operation time	MBI vs. MFI	Downgraded	Downgraded	No downgraded	No downgraded	Very low
	MBI vs. RAHI	Downgraded	Downgraded	No downgraded	No downgraded	Very low
	MBI vs. VI	Downgraded	Downgraded	No downgraded	No downgraded	Very low
	MFI vs. RAHI	Downgraded	Downgraded	No downgraded	No downgraded	Very low
	MFI vs. VI	Downgraded	Downgraded	No downgraded	No downgraded	Very low
	RAHI vs. VI	Downgraded	Downgraded	No downgraded	No downgraded	Very low
drainage volume	MBI vs. MFI	Downgraded	Downgraded	No downgraded	No downgraded	Very low
	MBI vs. RAHI	Downgraded	Downgraded	No downgraded	Downgraded	Very low
	MBI vs. VI	Downgraded	Downgraded	No downgraded	No downgraded	Very low
	MFI vs. RAHI	Downgraded	Downgraded	No downgraded	No downgraded	Very low
	MFI vs. VI	Downgraded	Downgraded	No downgraded	No downgraded	Very low
	RAHI vs. VI	Downgraded	Downgraded	No downgraded	No downgraded	Very low
permanent facial nerve palsy	MBI vs. MFI	Downgraded	Downgraded	No downgraded	No downgraded	Very low
	MBI vs. RAHI	Downgraded	Downgraded	No downgraded	No downgraded	Very low
	MBI vs. VI	Downgraded	Downgraded	No downgraded	No downgraded	Very low
	MFI vs. RAHI	Downgraded	Downgraded	No downgraded	No downgraded	Very low
	MFI vs. VI	Downgraded	Downgraded	No downgraded	No downgraded	Very low
	RAHI vs. VI	Downgraded	Downgraded	No downgraded	No downgraded	Very low
tumor size	MBI vs. MFI	Downgraded	Downgraded	No downgraded	No downgraded	Very low
	MBI vs. RAHI	Downgraded	Downgraded	No downgraded	No downgraded	Very low
	MBI vs. VI	Downgraded	No downgraded	No downgraded	No downgraded	Low
	MFI vs. RAHI	Downgraded	Downgraded	No downgraded	No downgraded	Very low
	MFI vs. VI	Downgraded	No downgraded	No downgraded	No downgraded	Low
	RAHI vs. VI	Downgraded	Downgraded	No downgraded	No downgraded	Very low
hematoma	MBI vs. MFI	Downgraded	Downgraded	No downgraded	No downgraded	Very low
	MBI vs. RAHI	Downgraded	Downgraded	No downgraded	No downgraded	Very low
	MBI vs. VI	Downgraded	Downgraded	No downgraded	No downgraded	Very low
	MFI vs. RAHI	Downgraded	Downgraded	No downgraded	No downgraded	Very low
	MFI vs. VI	Downgraded	Downgraded	No downgraded	No downgraded	Very low
	RAHI vs. VI	Downgraded	Downgraded	No downgraded	No downgraded	Very low
wound infection	MBI vs. MFI	Downgraded	Downgraded	No downgraded	No downgraded	Very low
	MBI vs. RAHI	Downgraded	Downgraded	No downgraded	No downgraded	Very low
	MBI vs. VI	Downgraded	Downgraded	No downgraded	No downgraded	Very low
	MFI vs. RAHI	Downgraded	Downgraded	No downgraded	No downgraded	Very low
	MFI vs. VI	Downgraded	Downgraded	No downgraded	Downgraded	Very low
	RAHI vs. VI	Downgraded	Downgraded	No downgraded	Downgraded	Very low
transient facial palsy	MBI vs. MFI	Downgraded	Downgraded	No downgraded	No downgraded	Very low
	MBI vs. RAHI	Downgraded	Downgraded	No downgraded	No downgraded	Very low
	MBI vs. VI	Downgraded	Downgraded	No downgraded	No downgraded	Very low
	MFI vs. RAHI	Downgraded	Downgraded	No downgraded	No downgraded	Very low
	MFI vs. VI	Downgraded	Downgraded	No downgraded	No downgraded	Very low
	RAHI vs. VI	Downgraded	Downgraded	No downgraded	No downgraded	Very low
salivary complications	MBI vs. MFI	Downgraded	Downgraded	No downgraded	No downgraded	Very low
	MBI vs. RAHI	Downgraded	Downgraded	No downgraded	No downgraded	Very low
	MBI vs. VI	Downgraded	Downgraded	No downgraded	No downgraded	Very low
	MFI vs. RAHI	Downgraded	Downgraded	No downgraded	No downgraded	Very low
	MFI vs. VI	Downgraded	Downgraded	No downgraded	No downgraded	Very low
	RAHI vs. VI	Downgraded	Downgraded	No downgraded	No downgraded	Very low
**Frey syndrome**	MBI vs. MFI	Downgraded	Downgraded	No downgraded	No downgraded	Very low
	MBI vs. RAHI	Downgraded	Downgraded	No downgraded	No downgraded	Very low
	MBI vs. VI	Downgraded	Downgraded	No downgraded	No downgraded	Very low
	MFI vs. RAHI	Downgraded	Downgraded	No downgraded	No downgraded	Very low
	MFI vs. VI	Downgraded	Downgraded	No downgraded	No downgraded	Very low
	RAHI vs. VI	Downgraded	Downgraded	No downgraded	No downgraded	Very low

Based on all the above information, we GRADEd each network estimate according to the following criteria.

(1) Study limitations: We downgraded by evidence at high risk of bias.

(2) Imprecision: We considered a clinically meaningful threshold for OR to be 0.80 or 1.25 and downgraded the estimate if the OR point estimate is 1 or more and the lower limit of its CrI is below 0.80; or if the OR point estimate is less than 1 and the upper limit of its CrI is above 1.25. We downgraded when the CrI of MD included zero between the upper and lower CrI limits.

(3) Inconsistency: We looked at the results of node-splitting and we downgraded the comparisons with important inconsistency (p<0.05), where we have not downgraded for imprecision.

(4) Indirectness: We downgraded singly-connected nodes for indirectness because evaluation of transitivity for such nodes is unclear.

(5) Publication bias: Publication bias could not be assessed as there were <10 trials available for each of the comparisons.

## 4 Discussion

This is the first systematic review and network meta-analysis to compare the surgical outcomes and complications of four major parotidectomy incisions. There were no significant differences in operation time, drainage volume, or permanent facial palsy. Similarly, no differences were found in salivary complications, wound infection, hematoma, or Frey syndrome. The incision satisfaction score was statistically significant in the comparison between the VI and MBI. Moreover, the VI had a smaller tumor size than MBI and MFI, and MFI had a significantly lower risk of transient facial palsy than MBI.

Generally, the following considerations govern the surgical incision design: full surgical field exposure and operability of lesion resection. Based on the above principles, the traditional Blair incision was gradually established as the most common parotidectomy method after modification. Unfortunately, these S-shaped incisions may leave a visible scar on the face and cause psychological distress (33). People are paying more attention to the requirements of beauty as their quality of life improves, and medical research has begun to investigate the potential for a good aesthetic effect while maintaining safety (5). The outcomes of primary incisions have been widely discussed; however, there has been a lack of convincing evidence on which procedure is optimal. Relevant RCTs and pairwise meta-analyses are few and far between, and previously published observational studies have had inconsistent results, which may be related to heterogeneity in the population and surgical technique. We performed a comprehensive Bayesian network meta-analysis to compare the outcomes of major surgical incisions in parotidectomy.

Scarring after parotidectomy is estimated to be between 54% and 60% based on prior research (34, 35). Traditional MBI incisions inevitably leave scarring on the face and neck, and numerous studies have noted significant patient dissatisfaction with scarring, which affects long-term quality of life (36). Although some techniques, such as skin flap and fascia reconstruction, can help prevent this issue, locating a better concealed incision can also help lessen the cosmetic negative impacts of scars (37). The novel VI, consisting of only anterior and posterior auricular incisions, has shown good cosmetic results in some previous studies (27, 31, 38). Similarly, based on the results of the SUCRA, our study rated VI as an approach with a higher incision satisfaction score and found it significantly superior to MBI. Furthermore, VI was significantly associated with tumor size when compared with MBI and MFI, indicating that tumor size is one of the parameters used by surgeons to choose surgical incisions. However, SUCRA data revealed that VI required the most operation time and MBI the least, despite no statistically significant difference between the four incisions. This might be attributed to a higher level of mastery of classical procedures; therefore, as proficiency increases, the surgical times for emerging incisions can be expected to decrease.

Owing to the substantial blood supply in the parotid gland region, much of the leaking blood, as well as saliva released by the remaining gland, would be collected in the cavity generated following parotidectomy (39). The presence of these fluids can cause complications such as seroma, and head and neck wounds should be drained with a drainage tube, according to the national consensus (39). Excessive drainage flow is likely to cause complications, such as infection and salivary fistula, resulting in prolonged hospital stays and increased medical costs (40). Notably, although there was no statistically significant difference in drainage volume between the four types of incisions, the RAHI was classified in the SUCRA ranking as the incisions with the least drainage.

Transient facial palsy is the most frequent early complication of parotidectomy (41). Permanent facial palsy after parotidectomy is the most serious complication that affects patients’ quality of life. According to previous studies, the incidences of early transient facial palsy and long-term permanent facial palsy are 42-45% and 0-3.9%, respectively (34, 35). With the gradual standardization of parotid surgery for the dissection and protection of facial nerves, and the wide application of intraoperative facial nerve monitoring, the incidence of facial paralysis has been greatly reduced; this in turn reduces the difference in the incidence of facial paralysis caused by different incisions (42, 43). In this review, the SUCRA results showed that conventional MBI had the highest risk of facial palsy. Furthermore, MBI’s SUCRA ranking in complications such as hematoma, wound infection, and salivary gland damage remained low, although the difference was not statistically significant in the pooled comparison results.

Frey syndrome occurs in 4-62% of patients 6 to 18 months after parotidectomy and is characterized by gustatory sweating and flushing (44). The incidence of Frey syndrome was documented in 12 of the included studies; however, none of these studies described the use of objective methods for diagnosis, which may result in the reported incidence being lower than the true value (45). A number of surgical techniques have been described to prevent this complication, and studies have attempted to determine the best way to reduce its incidence (46). Studies have shown that the size of the parotid gland tumor affects the incidence of Frey syndrome, which is explained by the fact that the less parotid tissue that needs to be dissected, the lower the likelihood of parasympathetic supply disruption (18, 47, 48). This view is seemingly supported by our findings, which shows that the SUCRA ranking of Frey syndrome and tumor size are consistent among the four incisions.

Although our meta-analysis yielded several novel results, it had certain limitations. First, this study contained only one RCT, with the remainder being single-center observational studies with potential selection and reporting bias. Second, two-thirds of the studies were conducted in East Asia, limiting the worldwide generalizability of our findings. Third, there was significant heterogeneity between MBI and the other three incisions in the pairwise meta-analyses. Fourth, some of the primary outcomes were rated as low or very low in evidential strength, based on the GRADE evaluation. Therefore, this may undermine the strength of the current findings. However, it is important to understand that high-quality evidence might be difficult to obtain because grade judgments can be overly cautious (49).

## Conclusion

Based on published studies, our network meta-analysis provides updated evidence for the multiple outcomes of MBI, MFI, RAHI, and VI. The most important advantages of VI are good incision satisfaction and better performance in the management of complications. Further randomized controlled trials and complementary outcome data are needed to test the credibility of our findings.

## Data availability statement

The original contributions presented in the study are included in the article/**Supplementary Material**. Further inquiries can be directed to the corresponding author.

## Author contributions

All the authors worked together to complete the paper. YJ, SY, and CS did the literature search; YJ, SY, and YL formed the study design; Data collection was done by SY, YL, YH, and ZF. SY, ZF, and YL analyzed the data; BC, SS, JW and XW interpreted the data; YJ, SY, and CS wrote the manuscript; YJ, SY, and BC critically reviewed the manuscript. All authors contributed to the article and approved the submitted version.

## Funding

This project was supported by applied medicine research project of Hefei Health Committee (hwk2019zc004).

## Acknowledgments

We thank the original studies included for providing data for our study.

## Conflict of interest

The authors declare that the research was conducted in the absence of any commercial or financial relationships that could be construed as a potential conflict of interest.

## Publisher’s note

All claims expressed in this article are solely those of the authors and do not necessarily represent those of their affiliated organizations, or those of the publisher, the editors and the reviewers. Any product that may be evaluated in this article, or claim that may be made by its manufacturer, is not guaranteed or endorsed by the publisher.
