# Supplementary material for: Comparison of various surgical incisions in parotidectomy: A systematic review and network meta-analysis
Source: Front Oncol. 2022 Aug 5;12:972498. doi: 10.3389/fonc.2022.972498 (PMC9389557; doi:10.3389/fonc.2022.972498)
Supplement: Supplementary file 1 [file DataSheet_1.docx]

**search strategy:**

**Pubmed and Web of science**

((((((Blair incision) OR (periauricular incision)) OR (V-shaped incision)) OR (retroauricular hairline incision)) OR (rhytidectomy incision)) OR (facelift incision)) AND (((parotid) OR (parotidectomy)) OR (parotid surgery))

**Embase**

#1. 'blair incision'

#2. 'periauricular incision'

#3. 'v-shaped incision'

#4. 'retroauricular hairline incision'

#5. 'rhytidectomy incision'

#6. 'facelift incision'

#7. parotid

#8. parotidectomy

#9. 'parotid surgery'

#10. parotid OR parotidectomy OR 'parotid surgery'

#11. 'blair incision' OR 'periauricular incision' OR

'v-shaped incision' OR 'retroauricular hairline

incision' OR 'rhytidectomy incision' OR 'facelift

incision'

#12. (parotid OR parotidectomy OR 'parotid surgery')

AND ('blair incision' OR 'periauricular incision'

OR 'v-shaped incision' OR 'retroauricular

hairline incision' OR 'rhytidectomy incision' OR

'facelift incision')

**Cochrane**

#1 Blair incision

#2 periauricular incision

#3 V-shaped incision

#4 retroauricular hairline incision

#5 rhytidectomy incision

#6 facelift incision

#7 #1 OR #2 OR #3 OR #4 OR #5 OR #6

#8 parotid

#9 parotidectomy

#10 parotid surgery

#11 #8 OR #9 OR #10

#12 #7 AND #11
